# *In vivo* mechanotransduction: Effect of acute exercise on the metabolomic profiles of mouse synovial fluid

**DOI:** 10.1016/j.ocarto.2021.100228

**Published:** 2021-12-08

**Authors:** Alyssa K. Hahn, Rachel A. Rawle, Brian Bothner, Erika Barboza Prado Lopes, Timothy M. Griffin, Ronald K. June

**Affiliations:** a Molecular Biosciences Program, Montana State University, Bozeman, MT, 59717, USA; b Department of Cell Biology & Neuroscience, Montana State University, Bozeman, MT, 59717, USA; c Department of Biological and Environmental Sciences, Carroll College, Helena, MT, 59625, USA; d Department of Chemistry & Biochemistry, Montana State University, Bozeman, MT, 59717, USA; e Aging and Metabolism Research Program, Oklahoma Medical Research Foundation (OMRF), Oklahoma City, OK, 73104, USA; f Veterans Affairs Medical Center, Oklahoma City, OK, 73104, USA; g Department of Mechanical & Industrial Engineering, Montana State University, Bozeman, MT, 59717, USA

## Abstract

**Objective:**

Exercise is known to induce beneficial effects in synovial joints. However, the mechanisms underlying these are unclear. Synovial joints experience repeated mechanical loading during exercise. These mechanical stimuli are transduced into biological responses through cellular mechanotransduction. Mechanotransduction in synovial joints is typically studied in tissues. However, synovial fluid directly contacts all components of the joint, and thus may produce a whole-joint picture of the mechanotransduction response to loading. The objective of this study was to determine if metabolic phenotypes are present in the synovial fluid after acute exercise as a first step to understanding the beneficial effects of exercise on the joint.

**Material and methods:**

Mice underwent a single night of voluntary wheel running or standard housing and synovial fluid was harvested for global metabolomic profiling by LC-MS. Hierarchical unsupervised clustering, partial least squares discriminant, and pathway analysis provided insight into exercise-induced mechanotransduction.

**Results:**

Acute exercise produced a distinct metabolic phenotype in synovial fluid. Mechanosensitive metabolites included coenzyme A derivatives, prostaglandin derivatives, phospholipid species, tryptophan, methionine, vitamin D3, fatty acids, and thiocholesterol. Enrichment analysis identified several pathways previously linked to exercise including amino acid metabolism, inflammatory pathways, citrulline-nitric oxide cycle, catecholamine biosynthesis, ubiquinol biosynthesis, and phospholipid metabolism.

**Conclusion:**

To our knowledge, this is the first study to investigate metabolomic profiles of synovial fluid during *in vivo* mechanotransduction. These profiles indicate that exercise induced stress-response processes including both pro- and anti-inflammatory pathways. Further research will expand these results and define the relationship between the synovial fluid and the serum.

## 1 Introduction

Synovial joints allow force transmission and joint rotation between long bones. Synovial joints, such as the knee, hip, hands, and shoulder, are common sites of osteoarthritis (OA). Because synovial joints experience frequent mechanical loading during joint motion, the biomechanical properties and mechanobiological responses of tissues from these joints have been subject to extensive research.

Mechanotransduction is the conversion of a mechanical stimulus into a biological response. Within the synovial joint, the articular cartilage and periarticular bone have been most studied in the context of mechanotransduction. Chondrocytes, the highly specialized resident cell type in articular cartilage, produce and maintain the cartilage matrix by responding to the mechanical environment in the joint [1]. Studies show that physiological dynamic loading in the joint maintains the cartilage matrix by promoting anabolic processes in chondrocytes [2]. In contrast, cartilage matrix synthesis is reduced and breakdown is increased when loading is absent or exceeds the physiologic range, as occurs with high impact loads and tissue strains resulting from injury or overuse [3,4]. When chondrocytes fail to maintain homeostasis of these anabolic and catabolic processes, osteoarthritis (OA) may develop [5]. Physiological loading is also necessary to maintain bone homeostasis. A lack of physiological loading results in bone resorption, whereas enhanced physiological loading stimulates bone formation [6]. Osteoblasts and osteocytes are the main mechanosensitive cells in the bone that respond to mechanical stimuli by promoting bone growth, remodeling, and repair to maintain homeostasis of the bone [6].

Synovial fluid (SF) has received less focus in joint mechanotransduction studies despite it being a mechanosensitive fluid with non-Newtonian flow characteristics that bathes the articular cartilage and periarticular bone surfaces [7,8]. SF is a plasma dialysate that is secreted by the synovial membrane. It contains a pool of molecules originating in both the blood plasma and the joint. Numerous cells throughout the joint may secrete factors into the SF, including synoviocytes, chondrocytes, osteoblasts, osteoclasts, osteocytes, fibroblasts, resident and infiltrating immune cells, and cells from other connective joint tissues including ligaments, tendons, and menisci [8]. Consequently, SF is likely to reflect the overall local state of the joint. Although mechanotransduction studies have focused on the lubricating properties of SF, a metabolic analysis of SF may provide a whole-joint perspective on mechanotransduction in synovial joints.

Metabolomics, or the study of small molecules in a biological system, is a promising method to characterize changes in joint biology [9]. Changes in the metabolome occur on a much faster timescale than in the proteome or genome, and thus more sensitively reflect the physiological state of the system [10]. Global metabolomic profiling, specifically, is a large-scale approach that detects all possible metabolites in a biological system, as opposed to targeted approaches that focus on a specific predetermined subset [11]. Thus, a metabolomic analysis of the SF provides a unique approach to evaluate mechanosensitive pathways that are stimulated during joint loading.

Mechanotransduction in synovial joints has been of particular interest in regard to OA. OA is the most common degenerative joint and is most associated with the deterioration of articular cartilage, and clinical studies demonstrate that exercise and muscle strengthening can reduce symptoms of OA [[12], [13], [14], [15], [16]]. Despite these empirical data, the mechanisms underlying the beneficial effects of exercise on joint health remain unclear.

While there is evidence that exercise has beneficial effects in the management of OA, no study to date has evaluated the effect of exercise on global joint metabolism. To take the first steps in understanding how exercise affects the joint metabolome, we examined metabolomic perturbations in SF following a single bout of nightly voluntary wheel running in young adult healthy male mice. Control sedentary animals were housed under standard cage conditions. Global metabolomic profiling of SF was conducted by untargeted LC-MS analysis. We aimed to identify specific mechanosensitive metabolites and mechanosensitive pathways in the SF to provide insight into the overall joint response to acute exercise. For this manuscript we use a broad definition of mechanosensitive to include metabolites induced either by direct mechanical loading of relevant joint tissues or indirectly through the systemic effects of exercise. We hypothesized that acute exercise would decrease inflammatory pathways and increase anabolic pathways associated with joint homeostasis.

## 2 Methods

### 2.1 Experimental design

Four separate litters of 14-week-old male C57BL/6J mice were obtained from a breeding colony at the Oklahoma Medical Research Foundation (OMRF). Mice from each litter were randomly assigned to the control or exercise groups. The control group (control: n ​= ​6) was single-housed for one night in standard caging with a hut and bedding but no wheel. The exercised group (ex: n ​= ​8) was single-housed in a standard cage that included bedding and a stainless-steel mouse running wheel (diameter ​= ​11.5 ​cm, width ​= ​5.2 ​cm, Mini Mitter, Bend, OR). Wheel running data were automatically collected via magnetic reed switch sensors that count the number of wheel revolutions per minute as previously described [17]. Mice were placed in their respective cages between 4:30–4:45PM and removed between 7:30–8:00AM the following day. All experiments were conducted within the OMRF vivarium, which provides temperature-controlled rooms maintained at 22 ​± ​3 ​°C on 14/10-h light/dark cycles (8:00PM off/6:00AM on) with *ad libitum* access to food and water. All litters of animals were tested within a 3-week period, and each litter contributed 1–2 animals per experimental group. Litters were tested on separate days so that no more than 4 animals were tested at a given time. Animals were euthanized between 8:00–9:30AM, alternating between control and exercise groups. Animals were euthanized under isoflurane anesthesia by cardiac puncture and exsanguination. All animal procedures were conducted in accordance with a protocol approved by the Institutional Animal Care and Use Committee at OMRF.

### 2.2 Synovial fluid collection

Immediately after euthanasia, SF was recovered from the knee joint using a calcium sodium alginate compound (CSAC) as previously described [18]. The patellar tendon was transected superior to the patella and slightly retracted distally to allow a 2 ​mm diameter biopsy punch of Melgisorb (Tendra, REF 250600; Goteborg, Sweden) wound dressing to be inserted into the joint space using fine forceps. The joint was manually articulated for 10 ​s to absorb the SF. The wound dressing was then carefully removed and placed in an Eppendorf tube. 35 ​μL of Alginate Lyase in H_2_O (1 unit/mL concentration; derived from *Flavobacterium*, Sigma-Aldrich A1603-100 ​MG) was added to the tube, vortexed, and digested at 34 ​°C for 30 ​min. The viscosity was lowered by adding 15 ​μL of 1.0 ​M sodium citrate (C_6_H_5_Na_3_O_7_) to chelate the Ca^2+^ ions. The samples were measured for total volume and then frozen at −80 ​°C. Frozen samples were shipped on dry ice to the June Lab at Montana State University and maintained at −80 ​°C until metabolite extraction and analysis.

### 2.3 Metabolite extraction

Metabolites were extracted from SF as previously described with slight modification [19,20]. Thawed SF samples were centrifuged at 4 ​°C at 500×*g* for 5 ​min to remove cells and debris. The supernatant was collected and vacuum concentrated for ∼2 ​h. The dried pellet was re-suspended in mass spectrometry grade 50:50 water: acetonitrile at −20 ​°C for 30 ​min to extract metabolites. The sample was then vortexed for 3 ​min and centrifuged at 16,100×*g* for 5 ​min at 4 ​°C. Proteins were precipitated by adding 250 ​μL of acetone, followed by 3 ​min of shaking and overnight refrigeration at 4 ​°C. The mixture was then centrifuged for 5 ​min at 16,100×*g* for 5 ​min and supernatant was collected and vacuum concentrated. The dried pellet was re-suspended in 50:50 water:acetonitrile.

### 2.4 Analysis by HPLC-MS

Metabolite extracts were analyzed for metabolomic profiling using an Agilent 1290 UPLC system (Agilent, Santa Clara, CA) coupled to an Agilent 6538 Q-TOF mass spectrometer (Agilent Santa Clara, CA) in positive mode with a resolution of ∼20,000 and accuracy of ∼5 ​ppm. Samples were run on a Cogent Diamond Hydride HILIC 150 ​× ​2.1 ​mm column (MicroSolv, Eatontown, NJ) in normal phase using previously optimized gradient elution methods [19]. Total run time was 15 ​min including a 2-min wash prior to each sample.

## 3 Data processing

Raw data were converted from Agilent's proprietary file format (.d files) to mzXML files using Agilent MSConvert prior to being imported to MZMine 2.14 for data processing [21]. A noise level threshold of 1000 was applied. Retention times and mass-to-charge ratios (*m*/*z*) were normalized across spectra with a retention time tolerance of 0.25 ​min and *m*/*z* tolerance of 0.01 ​*m*/*z* or 30 ​ppm and peaks were aligned. Peaks were detected with a minimum time span of 0.1 ​min, minimum height of 1000, and *m*/*z* tolerance of 0.1 ​*m*/*z* or 30 ​ppm. Metabolite features with median intensity values of zero in both control and exercised groups were eliminated and any remaining zeroes were replaced with one-half the minimum peak intensity for further analysis [22]. For analysis, metabolite features were identified by their experimentally measured mass-to-charge (*m*/*z*) ratios. Common adducts formed during electrospray ionization include Na+ and H+ adducts. Mass-to-charge ratios were matched to putative metabolite identities using the metabolite database, METLIN (adducts: H^+^ and Na^+^; mass tolerance: 15 ​ppm; removed drugs and toxins) [23,24].

### 3.1 Metabolomic profiling and statistical analyses

Statistical analyses were performed in MetaboAnalyst [22] and MATLAB (Mathworks, Inc.). Raw data were normalized by the median, log transformed, standardized (mean-centered and divided by standard deviation), and filtered by standard deviation. Normalized data were used for all analyses unless otherwise noted, and data were checked to meet the relevant assumptions of the specific statistical tests. Kolmogorov-Smirnov (KS) tests were used to compare the median metabolite distributions of control and exercised datasets (*a priori* p_ks_<0.05). To identify differentially expressed metabolite features, fold changes (FC) were calculated (on data prior to normalization to compare the absolute differences, which can be skewed by normalization). To preserve the magnitude of the differences, fold changes were calculated based on the raw mean metabolite intensities between the control and exercised groups. Data were then normalized to the median on a per-metabolite basis and Student's T-tests and volcano plot analysis were applied. Enrichment analysis of differentially expressed metabolite features was performed using the *MS Peaks to Pathways mummichog* application in MetaboAnalyst [22,25]. *Mummichog* matches all detected metabolite features to potential metabolites (mass tolerance: 0.1 ​ppm) in the global network of pathways and searches for local pathway enrichment to identify the pathway activity and most likely metabolite identifications. *Mummichog* reports the activity network as pathways and calculates an FDR-corrected p-value to reveal the most likely enriched pathways (FDR-corrected p-value<0.05).

Hierarchical clustering analysis (HCA), clustergrams, and partial least squares-discriminant analysis (PLS-DA) were used to visualize both sample-to-sample variation and overall metabolomic profiles of control and exercised SF. PLS-DA score plots include 95% confidence ellipses to assess separation between the metabolomic profiles of control and exercised mice based on the first 2 components [26]. Mechanosensitive metabolites were identified using the variable of importance projection (VIP) scores from PLS-DA, correlation coefficients, and volcano plot analysis. VIP scores indicated which metabolite features contributed the most to the variation between exercised and sedentary groups.

To identify strongly correlated metabolite features we calculated pointwise biserial correlation coefficients, r_pb_. These were calculated on a per-metabolite basis between the intensities of the control and exercise groups. These correlations are a measure of the effect size of the median metabolite intensities between controls and exercised [27]: correlation values greater than zero represent metabolites with higher intensities in the exercised group and correlation values less than zero represent metabolites with higher intensity in the control group. We visualized these correlations by plotting the empirical cumulative distribution.

Lastly, significantly up- and down-regulated metabolite features revealed by volcano plot analysis (greater than twofold change and p-value<0.05) were considered mechanosensitive metabolites. Mechanosensitive metabolites were putatively identified using the metabolite database, METLIN (adducts: H^+^ and Na^+^; mass tolerance: 15 ​ppm; removed drugs and toxins) [23,24].

## 4 Results

**Fig. 1:**
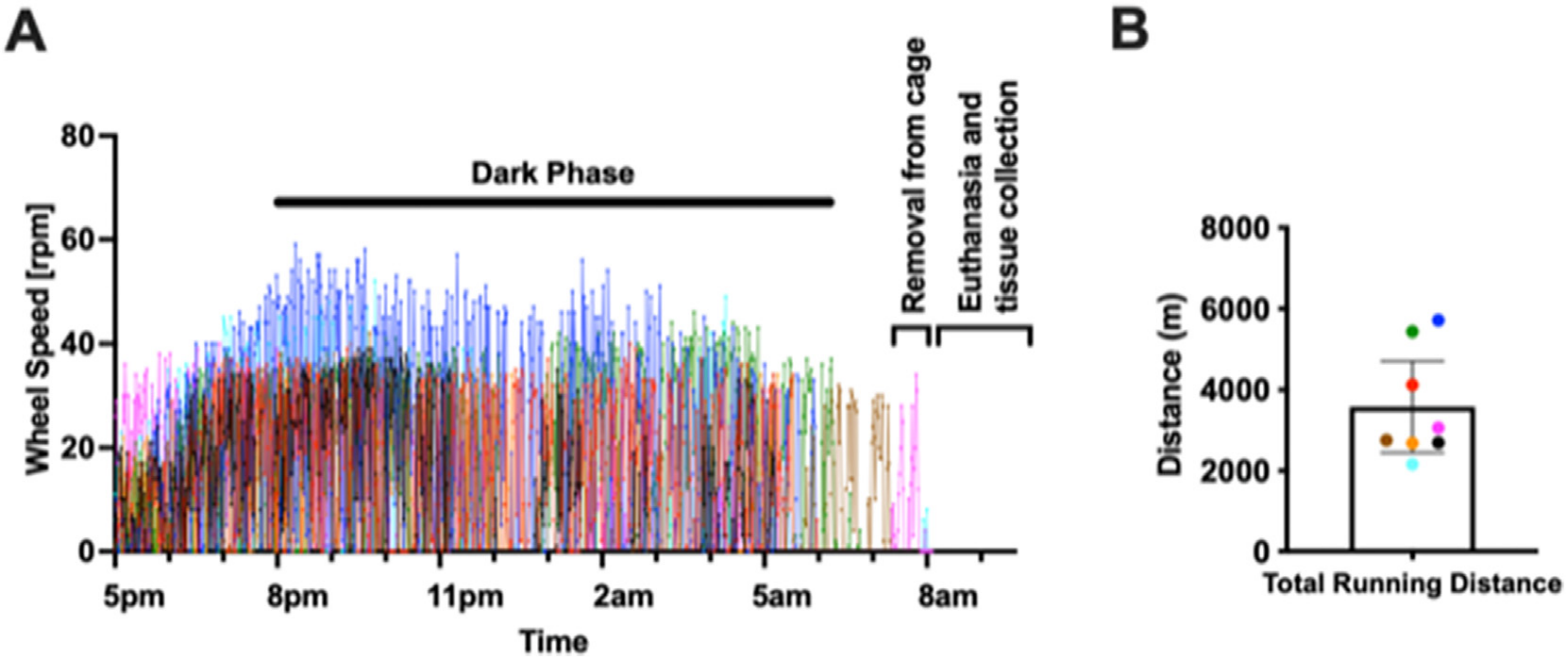
Acute exercise in mice. Mice selected for the exercise group (n ​= ​8) were individually housed in cages equipped with a stainless-steal running wheel (diameter ​= ​11.5 ​cm) between approximately 4:30pm to 7:30am the following morning. (A) Wheel speed in revolutions per minute (rpm) plotted against time. Each color denotes a separate animal, and dark phase refers to the time during which the room lights were turned off. (B) Total running distance in meters was calculated as the sum of the total revolutions from 5pm until removal from the cage multiplied by the wheel circumference. Each colored symbol refers to an individual animal as indicated in panel A. Value equals mean ​± ​95% CI.

The objective of this study was to determine the whole-joint metabolic response to acute exercise by profiling changes in the synovial fluid. On average, Ex animals voluntarily ran 3,581 ​m between 5pm and 8am [95%CI: 2450–4,712 ​m], prior to SF collection (Fig. 1). Most animals greatly reduced their running activity when the room lights came on at 6am, although some animals continued low levels of running until removal (Fig. 1).

### 4.1 Acute exercise altered global metabolomic profiles

**Fig. 2:**
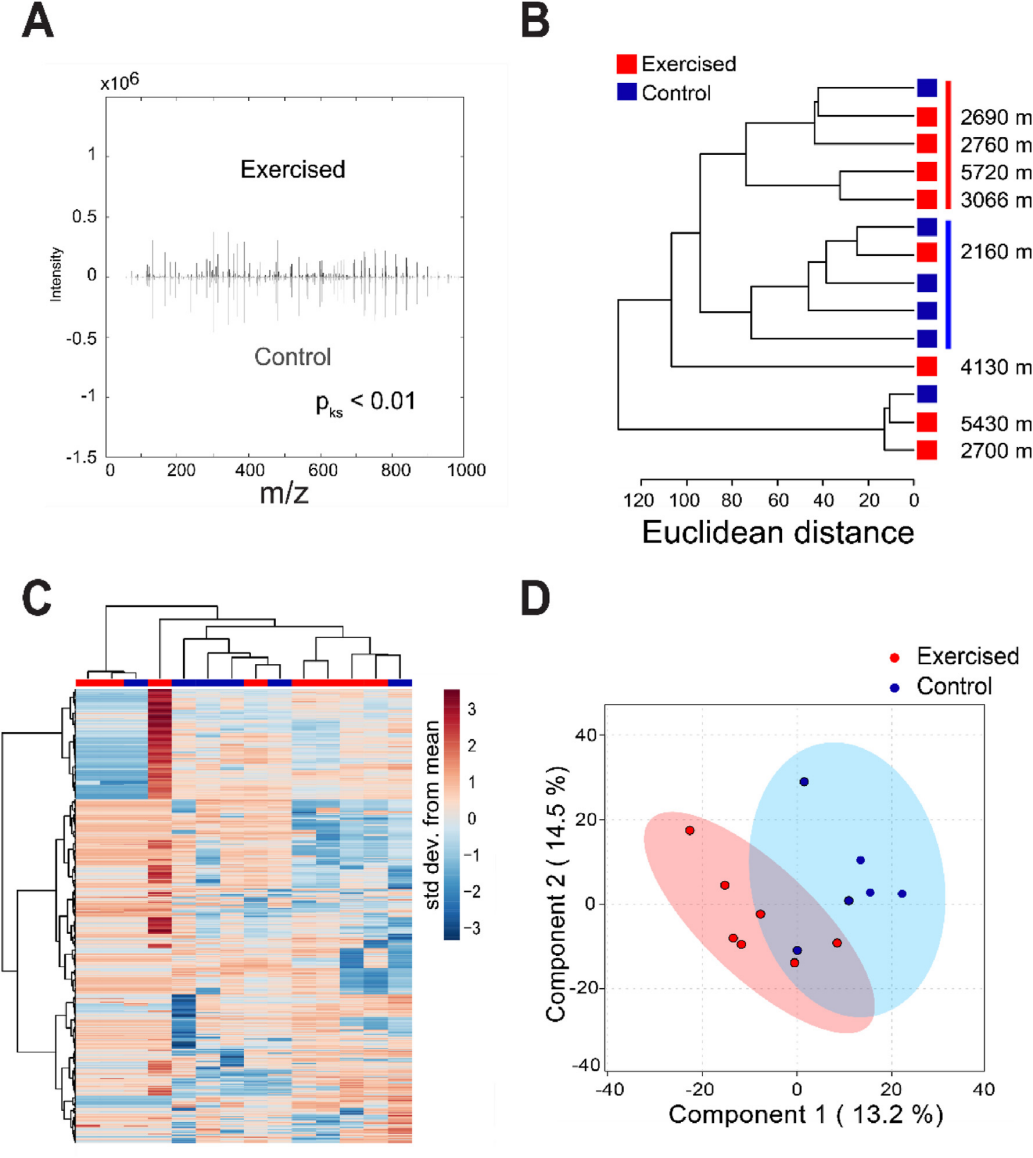
Acute exercise results in distinct metabolomic profiles. (A) Acute exercise alters the cumulative metabolite distributions. Mirrored metabolite feature distributions of *m*/*z* values and corresponding intensities were plotted to illustrate any differences in distributions. KS test showed a statistically significant (p_ks_<0.01) change in metabolite distributions comparing control to acute exercised SF. (B) Unsupervised hierarchical cluster analysis illustrates unsupervised clustering of most exercised (red line) and control samples (blue line). Tree distances are represented as Euclidean distances. Distances run by exercised mice shown on the right. Tree distances are represented as Euclidean distances. Red represents exercised SF and blue represents control SF. (C) Clustergram representation of all metabolite features detected shows overall metabolomic profiles of each sample. Clustering of samples is illustrated by the dendrogram on the top, and clustering of co-metabolites is illustrated by the dendrogram on the side. (D) Supervised PLSA-DA clearly discriminated between acute exercised and control samples. PLS-DA score plot illustrated separation between of exercised and control samples, with PC1 containing 13.2% of the variation and PC2 containing 14.5% of the variation.

A total of 2812 metabolite features were detected in mouse SF in this study. These features were used to generate global metabolomic profiles for exercised and sedentary cohorts. These analyses revealed that acute exercised induced substantial metabolomic changes in the SF. The global metabolomic profile of acute exercised SF was significantly different than sedentary SF (p_ks_<0.01; Fig. 2A). Unsupervised HCA assessed grouping of samples and calculated distances between clusters. This distinguished the majority of exercised samples from controls (Fig. 2B). However, HCA did not perfectly distinguish control from exercised samples with one sample of exercised SF falling within a cluster of four control samples. This exercised sample was from the mouse that ran the least of all mice in this study (Fig. 2B). Clustergram analysis found groups of co-regulated metabolites and inter-sample variation that likely contributes to the overall clustering. The distinct metabolomic profiles between exercised and sedentary controls further supports the SF as a mechanosensitive fluid, with a single bout of physical activity inducing metabolic changes in the SF.

A supervised clustering method, PLS-DA, was used to further examine the metabolomic profiles of acute exercised and control SF. PLS-DA clearly separated control from acute exercised SF samples with the first 2 components containing 27.7% of the variation between datasets (Fig. 2D). The 95% confidence ellipses show separation between exercised and control cohorts, with some overlap between cohort clusters (Fig. 2D). Taken together, KS-test, HCA, and PLS-DA suggest that acute exercise induced metabolic changes in the SF in comparison to unexercised controls, further supporting the SF as a fluid that captures changes associated with exercise.

### 4.2 Mechanosensitive metabolites

We investigated specific metabolite features associated with acute exercise in SF to gain insight into *in vivo* mechanotransduction in the joint. Variable Importance in Projection (VIP) in PLS-DA, correlation coefficient analysis, and volcano plots analysis were used to reveal mechanosensitive metabolites.

**Fig. 3:**
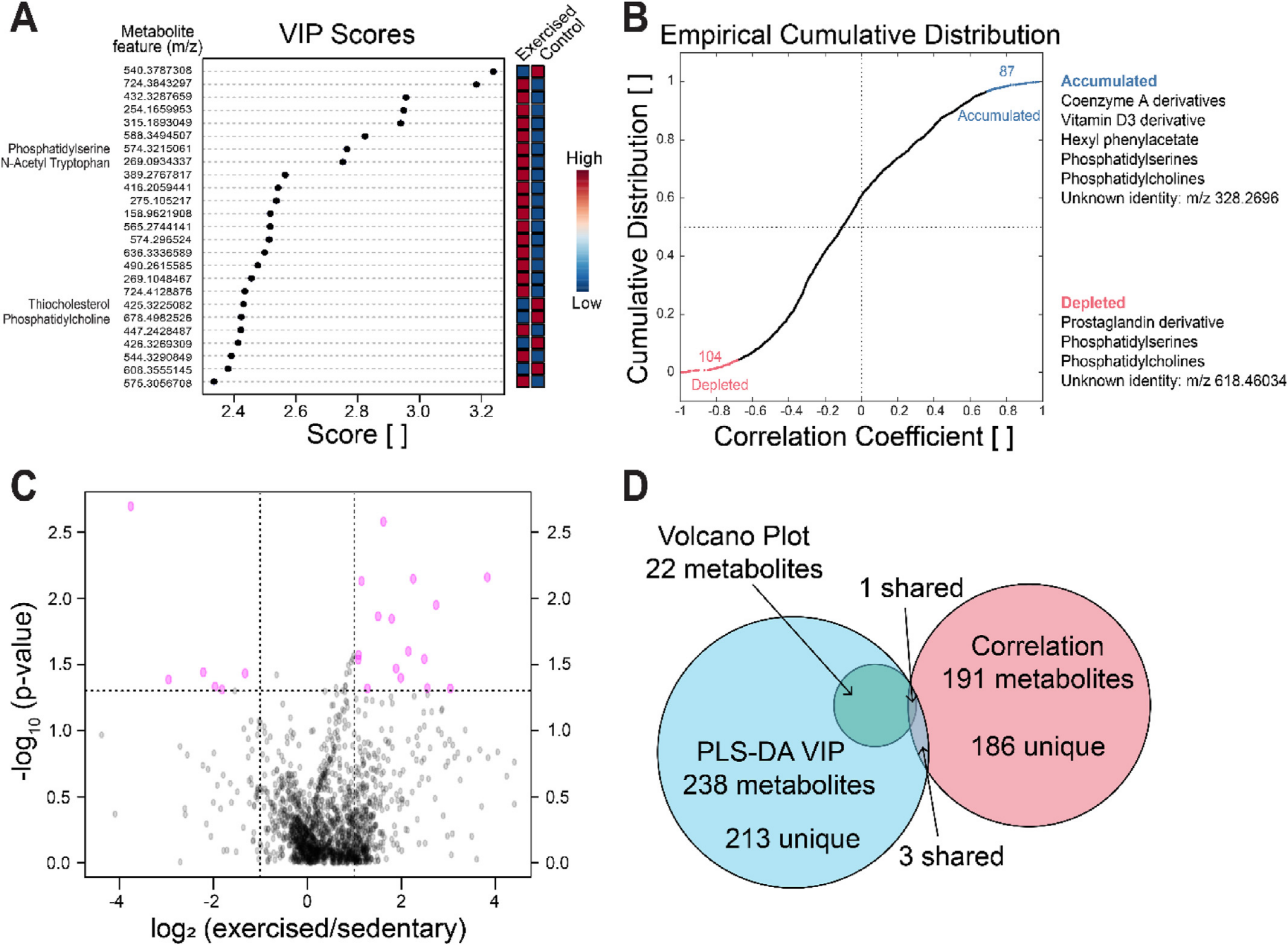
Mechanosensitive metabolites in mouse synovial fluid. (A) PLS-DA VIP scores determined the top 25 mechanosensitive metabolite features plotted by their *m*/*z* value that contributed the most to the variation between datasets. The majority of metabolite features with high VIP scores were higher in exercised than controls. These features are matched to metabolite identities in Table 1. Select mechanosensitive metabolite features higher in exercised than control SF matched with metabolite identities N-acetyl tryptophan and phosphatidylserine. (B) Cumulative distribution of pointwise biserial correlations for metabolite features correlated to acute exercise. Features with a correlation coefficient greater than 0.7 or less than −0.7 were considered accumulated (blue) or depleted (red), respectively. The top 10 accumulated and top 10 depleted metabolite features were considered mechanosensitive metabolites and matched with metabolite identities in Table 2 (C) Volcano plot analysis shows metabolite features significantly upregulated or downregulated by exercise. Metabolite features in the upper right and left regions (dashed lines) have a p-value<0.05 and greater than twofold change. The significant metabolite features are matched to metabolite identities in Table 3 (D) Venn Diagram showing metabolites used in fold-change analysis. There was substantial overlap of metabolites from the volcano plot and VIP analysis. However, there was limited overlap between the metabolites used from the correlation analysis. Note *m*/*z* ratios (mass-to-charge) may match multiple metabolite identities depending on adduct formation (H+ or Na+) occurring during ionization.

VIP in PLS-DA found metabolites that contribute the most to the separation between exercised and control cohorts in the PLS-DA score plot (Fig. 2D; Fig. 3A). Metabolite features with the highest scores have the greatest ability to classify samples into their respective cohorts. Phosphatidylcholine (PC), phosphatidylserine (PS), thiocholesterol, and tryptophan derivatives were some of the putatively identified mechanosensitive metabolites (Table 1, Supplemental Table 4). Interestingly, 20 of 25 metabolites with the highest VIP scores had metabolite intensities greater in exercised than control SF (Fig. 3A, Table 1) Supplemental Table 4. This indicates that the differences between exercised and sedentary control SF are mainly attributed to exercised-induced increases in metabolite expression as opposed to decreases.

**Table 1:** Mechanosensitive metabolites identified by PLS-DA VIP scores. VIP scores in PLS-DA identified metabolite features that contributed the most to the variation between control and exercised datasets. The top 25 metabolite features were matched with potential metabolite identities using the metabolite database METLIN (adducts: H^+^, Na^+^; mass tolerance: 15 ​ppm). False positives and drugs were removed from compound matches. An uncondensed list of compound matches is shown in Supplemental Table 4. Mass-to-charge ratios may match multiple metabolite identities because of either isoptomers or differential adduct formation (H+ or Na+) during ionization. Columns: Metabolite identity-putative metabolite match between experimental data and Metlin database. Mass-mass of putative metabolite. PPM—difference between database and experimental metabolite in parts per million. Adduct—ion adduct of experimental metabolite feature. Feature—mass of experimental metabolite feature. VIP score—variable importance projection score for PLS-DA analysis. High/Low—arrows indicate if the expression increased or decreased with exercise.

MECHANOSENSITIVE METABOLITE FEATURES						
PLS-DA VIP						
Metabolite Identity	Mass	PPM	Adduct	Feature	VIP score	High (↑)/Low (↓) with Ex
				540.3787308	3.2385	↓
				724.3843297	3.1837	↑
				432.3287659	2.9562	↑
				254.1659953	2.9487	↑
alpha-licanic acid	292.2038	11	Na	315.1893049	2.9388	↑
Oxo-phytodienoic Acid	292.2038	11				
Colnelenic acid	292.2038	11				
Octadecatrienoic acid derivatives	292.2038	11				
Etherolenic acid	292.2038	11				
			Na	588.3494507	2.8234	↑
Phosphatidylserine	573.3067	13	H	574.3215061	2.7651	↑
Phenyalanyl-cysteine	268.0882	7	H	269.0934337	2.7517	↑
benzopyrene derivatives	268.0888	9				
3-hydroxyphenoytoin	268.0848	5				
5-(4-hydroxyphenyl-5-phenylhydantoin	268.0848	5				
2,4-Imidazolidinedione	268.0848	5				
N-acetyl-d-tryptophan	246.1004	14	Na			
Methionyl-prolnie/prolyl-methionine	246.1038	1				
				389.2767817	2.5654	↑
				416.2059441	2.5421	↑
(1xi,3S)-1,2,3,4-Tetrahydro-1-methyl-beta-carboline-1,3-dicarboxylic acid	274.0954	9	H	275.105217	2.5367	↑
p-(3,4-Dihydro-6-methoxy-2-naphthyl)phenol	252.115	3	Na			
(1Z,4Z)-1,5-bis(4-hydroxyphenyl)-1,4-pentadiene	252.115	3				
2-Phenylethyl 3-phenyl-2-propenoate	252.115	3				
Cinnamyl phenylacetate	252.115	3				
				158.9621908	2.5173	↑
Chaetoglobosin N	542.2781	12	Na	565.2744141	2.5167	↑
				574.296524	2.5135	↑
				636.3336589	2.4987	↑
				490.2615585	2.4765	↑
Idebenone metabolite (QS-4)	268.0947	10	H	269.1048467	2.4558	↑
diethyl 2,6-dimethyl-4-oxo-4h-pyran-3,5-dicarboxylate	268.0947	10				
3,3′-diindolylmethane	246.1157	0	Na			
Phosphatidylserine	701.4268	4	Na	724.4128876	2.4345	↑
Unoprostone isopropyl ester	424.3189	8	H	425.3225082	2.4303	↓
4,4′-Diaponeurosporene	402.3287	10	Na			
Vitamin D3 derivatives	402.3298	8				
Thiocholesterol	402.332	2				
Phosphatidylcholine	677.4996	12	H	678.4982526	2.4231	↓
clavulone derivatives	446.2305	11	H	447.2428487	2.4219	↑
6B-Hydroxybudesonide	446.2305	11				
ethyl-1-(2,4,6-trihydroxy-3-isobutyrylphenyl)butyl]-4-cyclohexene-1,3-dione	446.2304	11				
1-heptadecanoyl-sn-glycerol 3-phosphate	424.259	11	Na			
Phosphatidic acid	424.259	11				
				426.3269309	2.4128	↓
				544.3290849	2.391	↑
				608.3555145	2.3803	↓
2,3-diacetoxy-7,8-epoxy-24,29-dinor-1,3,5-friedelatriene-20-carboxylic acid	13	13	Na	575.3056708	2.3351	↑

We expanded our analysis of PLS-DA VIP scores from the top 25 metabolite features to all features with a VIP score greater than 1.5 to investigate pathways differences. Discriminative metabolite features were involved in corticosteroid, prostaglandin, and ubiquinol biosynthesis as determined by enrichment analysis (Supplemental Table 3).

Mechanosensitive metabolites that had the greatest accumulation or depletion with acute exercise were identified by correlation coefficient analysis. Correlation analysis examined the effects of acute exercise on metabolite intensities in comparison to controls. There were 191 statistically significant metabolite features that correlated with acute exercise, 87 of which were positively correlated (accumulated) and 104 were negatively correlated (depleted, Fig. 3B). The strongest positively and negatively correlated metabolites were considered mechanosensitive metabolites (Table 2, Supplemental Table 5). Of those metabolite features identified as mechanosensitive, putative metabolite identity matches included coenzyme A derivatives, a vitamin D3 derivative, a prostaglandin derivative, PS, and PC (Fig. 3B, Table 2, Supplemental Table 5).

**Table 2:** Mechanosensitive metabolites identified by pairwise biserial correlation coefficient analysis. Correlation coefficient analysis identified metabolite features that were accumulated or depleted after acute exercise. Strongly correlated features were matched with metabolite identities using METLIN (adducts: H^+^, Na^+^; mass tolerance: 15 ​ppm). False positives and drugs were removed from compound matches. An uncondensed list of compound matches is shown in Supplemental Table 5. Mass-to-charge ratios may match multiple metabolite identities because of either isoptomers or differential adduct formation (H+ or Na+) during ionization. Columns: Metabolite identity-putative metabolite match between experimental data and Metlin database. Mass-mass of putative metabolite. PPM—difference between database and experimental metabolite in parts per million. Adduct—ion adduct of experimental metabolite feature. Feature—mass of experimental metabolite feature. Coefficient—correlation coefficient. Accumulated/Depleted—indicates if the metabolite increased or decreased with exercise.

MECHANOSENSITIVE METABOLITE FEATURES						
Correlation Coefficient Analysis						
Metabolite Identity	Mass	PPM	Adduct	Feature	r_pb_	P-value
				491.4027405	−0.992	0.008
				618.4603464	−0.981	0.019
Phosphatidylcholine	591.39	9	Na	614.3850952	−0.969	0.006
				755.0263367	−0.967	0.033
Phosphatidylserine	511.3274	11	Na	534.3226522	−0.963	0.002
				928.7206607	−0.960	0.040
6-Monoacetyl morphine-3-O-glucuronide	373.2406	0	H	374.2474714	−0.959	0.002
Androsta-5,16-dienol [17,16-b] quinolin-3beta-ol	373.2406	0				
Dodecylphosphocholine	351.2538	11	Na			
2,3-dinor-6-keto Prostaglandin F1α-d9	351.2607	6				
				141.9570389	−0.956	0.011
				491.8885193	−0.954	0.046
				686.3826643	−0.952	0.003
				250.1348	0.929	0.023
				342.2328	0.934	0.020
Vitamin D3 derivative	522.3764	1	H	545.3847	0.944	0.056
				697.9982	0.948	0.052
				328.2565	0.949	0.004
Phenylglyoxylyl-CoA	899.1363	11	H	900.1329	0.961	0.039
3,4-didehydroadipyl-CoA semialdehyde	877.152	9	Na			
				328.2696	0.966	0.007
Phosphatidyserine	899.6615	9	H	900.6772	0.973	0.027
Phosphatidylcholine	877.6924	4	Na			
				359.2435	0.973	0.005
Hydroxypentobarbital	242.1267	2	H	243.133316	0.976	0.024
2,6-di-tert-butylbenzoquinone	220.1463	9	Na			
3-Phenylpropyl isovalerate	220.1463	9				
13-Nor-6-eremophilene-8,11-dione	220.1463	9				
Hexyl phenylacetate	220.1463	9				
2-Phenylethyl hexanoate	220.1463	9				
Tetradecene-1,3-diyne-diol derivatives	220.1463	9				
2-Methyl-1-phenyl-2-propanyl butyrate	220.1463	9				

Volcano plot analysis showed mechanosensitive metabolite features significantly upregulated (accumulated) and downregulated (depleted) based on p-value (before FDR-correction) and magnitude of fold change (Fig. 3C). 16 metabolite features were significantly upregulated and 6 were significantly downregulated (p-value<0.05, greater than twofold change; Fig. 3C). These mechanosensitive metabolite features were matched to putative metabolite identities shown in Table 3 (Supplemental Table 6). Select mechanosensitive features significantly upregulated by exercise included fatty acids, cysteinyl-phenylalanine, and tryptophan. Select mechanosensitive features significantly downregulated by exercise included vitamin D3 derivatives and thiocholesterol (Fig. 3C, Table 3, Supplemental Table 6).

**Table 3:** Mechanosensitive metabolites identified by volcano plot analysis. Mechanosensitive features upregulated or downregulated with exercise were identified by volcano plot analysis. Features with a greater than twofold change and p-value (prior to FDR correction) less than 0.05 were considered mechanosensitive metabolites. Metabolite identities were assigned via METLIN (adducts H^+^, Na^+^; mass tolerance: 15 ​ppm). False positives and drugs were removed from compound matches. An uncondensed list of compound matches is shown in Supplemental Table 6. Mass-to-charge ratios may match multiple metabolite identities because of either isoptomers or differential adduct formation (H+ or Na+) during ionization. Columns: Metabolite identity-putative metabolite match between experimental data and Metlin database. Mass-mass of putative metabolite. PPM—difference between database and experimental metabolite in parts per million. Adduct—ion adduct of experimental metabolite feature. Feature—mass of experimental metabolite feature. Fold Change—fold change of metabolite between exercise and control group. P-value—FDR-corrected p-value for comparing between exercise and control. Accumulated/Depleted—indicate if expression increased or decreased with exercise.

MECHANOSENSITIVE METABOLITE FEATURES							
Volcano Plot Analysis							
Metabolite Identity	Mass	PPM	Adduct	Feature	Fold Change	P-value	Accumulated (↑)/Depleted (↓)
				540.3787	0.07398	0.002015	↓
				724.3843	3.0682	0.002631	↑
				432.3288	14.191	0.006931	↑
				254.1660	4.7594	0.007132	↑
alpha-licanic acid	292.2038	11	Na	315.1893	2.2216	0.007407	↑
Colnelenic acid	292.2038	11					
Etherolenic acid	292.2038	11					
Octadecatrienoic acid derivatives	292.2038	11					
Oxo-phytodienoic acid derviatives	292.2038	11					
				588.3495	6.6728	0.01123	↑
Phosphatidylserine	573.3067	13	H	574.3215	2.8375	0.01366	↑
3-hydroxyphenytoin	268.0848	5	H	269.0934	3.4668	0.01427	↑
2,4-Imidazolidinedione, 5-(7-oxabicyclo [4.1.0]-hepta-2,4-dien-3-yl)-5-phenyl-	268.0848	5					
2,3-Dihydroxycarbamazepine	268.0848	5					
Benzo [a]pyrene derivatives	268.0888	9					
Cysteinyl-phenylalanine	268.0882	7					
Phenylalanyl-cysteine	268.0882	7					
N-acetyl-tryptophan	246.1004	14	Na				
methionyl-proline	246.1038	1					
prolyl-methionine	246.1038	1					
				389.2768	4.4307	0.02512	↑
				416.2059	2.1272	0.02681	↑
				158.9622	5.6077	0.02872	↑
				574.2965	2.1147	0.02902	↑
idebenone metabolite (QS-4)	268.0947	10	H	269.1048	3.6993	0.03387	↑
diethyl 2,6-dimethyl-4-oxo-4h-pyran-3,5-dicarboxylate	268.0947	10					
3,3′-diindolylmethane	246.1157	0	Na				
Unoprostone isopropyl ester	424.3189	8	H	425.3225	0.2154	0.03620	↓
4,4′-Diaponeurosporene	402.3287	10	Na				
fluorovitamin D3/fluorocholecalciferol derivatives	402.3298	8					
25-Fluorovitamin D3	402.3298	8					
Thiocholesterol	402.3298	8					
Phosphatidylcholine	677.4996	12	H	678.4983	0.3988	0.03687	↓
Phosphatidylethanolamine	677.4995	12					
				544.3291	3.9614	0.04001	↑
				608.3555	0.1292	0.04109	↓
				598.4739	0.2565	0.04653	↓
				291.2023	5.8616	0.04775	↑
				716.9248	2.4243	0.04794	↑
				782.4581	8.2303	0.04807	↑
Phosphatidylcholine	523.4002	6	Na	546.3927	0.2842	0.04888	↓
2-o-methyl PAF C18	523.4002	6					

Many mechanosensitive metabolite features overlapped among PLS-DA and volcano plot analyses, but none of these overlapped with the metabolites detected in the correlation analyses (Fig. 3D) A select few include phosphatidylserines, phosphatidylcholines, and vitamin D3 derivatives, which were identified in all three analyses. Thiocholesterol and a variety of fatty acids (*i.e.* octadecatrienoic acid derivatives) were identified by PLS-DA and volcano plot analysis. Taken together, these results suggest that phospholipids, vitamin D derivatives, and fatty acids are prominent mechanosensitive metabolites in the SF in response to acute exercise.

### 4.3 Mechanosensitive pathways

**Fig. 4:**
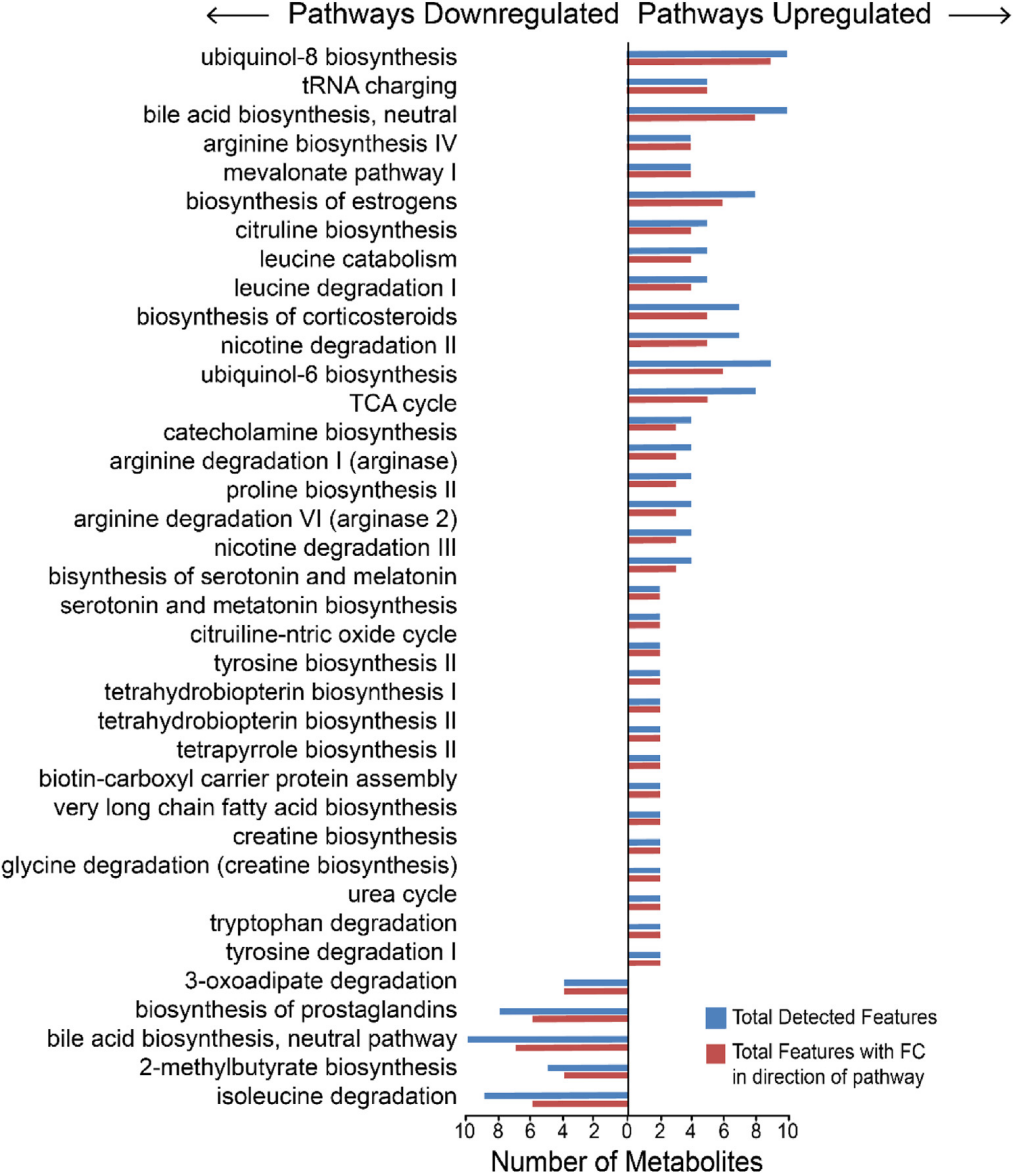
Acute exercise alters metabolic pathways in mouse synovial fluid. Enrichment analysis of metabolite features upregulated (right side) or downregulated (left side) following acute exercise identified significantly different biochemical pathways (FDR-corrected p-value<0.05). Blue bars represent all metabolite features detected in the dataset. Red bars represent metabolite features that were upregulated or downregulated corresponding to the directionality of the pathway (i.e. Leucine catabolim: a total of 9 metabolites in this pathway were detected but only 6 had a fold change less than one corresponding to the directionality of this pathway—downregulated)). Supplemental Tables 1 and 2 show the full lists of perturbed pathways.

To generate a metabolomic phenotype of SF following acute exercise, the computational algorithm, *mummichog*, was employed to predict functional pathway activity from the detected metabolite features [25]. Pathway analysis of metabolite features with higher intensities (FC ​> ​1) after acute exercise mapped to 33 pathways (FDR-corrected p-value<0.05) including ubiquinol (antioxidant) biosynthesis, tRNA charging, amino acid biosynthesis (arginine, proline, leucine, and tyrosine), citrulline and nitric oxide metabolism, the urea cycle, creatine biosynthesis, catecholamine biosynthesis, and fatty acid biosynthesis. Metabolite features with lower intensities after exercise (FC ​< ​1) mapped to 5 significant pathways (FDR-corrected p-value<0.05) including 3-oxoadipate degradation, biosynthesis of prostaglandins (inflammatory mediator), bile acid biosynthesis, 2-methylbutyrate biosynthesis, isoleucine degradation, and ketone oxidation (Fig. 4, Supplemental Tables 1-2). Taken together, these pathways show that the synovial fluid of exercised mice captures metabolites related to amino acid synthesis, as well as both pro- and anti-inflammatory pathways that indicate a complex response to exercise.

## 5 Discussion

To our knowledge, this is the first description of global metabolomic changes measured in SF after acute exercise. We measured changes in joint metabolism following acute exercise using global metabolomic profiling of SF from mice undergoing one night of voluntary wheel running. We detected a total of 2812 metabolite features in mouse SF. Multiple analyses (*i.e.* KS test, PLS-DA, and HCA) showed distinct metabolomic profiles between exercised and control SF samples. These results demonstrate that SF captures the response to exercise and associated mechanical loads. In the SF, changes in joint metabolism occur within hours of a diurnal bout of increased physical activity. Importantly, the SF likely captures changes in metabolites produced by synovial joint mechanosensitive cells as well as systemic exercise responses that diffuse into the SF.

Mechanosensitive metabolites were identified using VIP scores from PLS-DA, correlation coefficient analysis, and volcano plot analysis. Mechanosensitive metabolites included coenzyme A derivatives, prostaglandin derivatives, phospholipid species, tryptophan, methionine, vitamin D3, fatty acids, and thiocholesterol. Enrichment analysis identified many pathways previously linked to exercise including amino acid metabolism, inflammatory pathways, the citrulline-nitric oxide cycle, catecholamine biosynthesis, ubiquinol biosynthesis, and phospholipid metabolism.

Global metabolomic profiling detects small-molecules including substrates, co-factors, and other cytosolic molecules to generate a high-dimensional phenotype of a biological system. SF metabolomic profiles describe a comprehensive whole-joint phenotype generated by molecules potentially originating from multiple cell types across the joint [8]. The present data provide a biochemical profile of mechanosensitive metabolites involved in the overall joint response to a single night of wheel-running exercise in mice. These data provide an initial view of how exercise promotes healthy joint metabolism and how exercise therapy may function mechanistically as an intervention for OA joints to alleviate OA symptoms. It is important to recognize, however, that the results from a single exercise bout may not reflect the changes that occur following chronic, repeated bouts of exercise.

The metabolic phenotype generated in this study sheds light on the effects of acute exercise and its contribution to maintaining overall joint health. Acute exercise upregulated amino acid metabolism (arginine, proline, leucine, and tyrosine) in mouse SF. The SF is in direct contact with the articular cartilage—a known mechanosensitive tissue that relies on mechanical stimuli to maintain a healthy matrix [1,2]. The articular cartilage is an avascular tissue so chondrocytes rely on the SF for nutrients. Previous studies have found increased amino acid metabolism in primary chondrocytes in response to short-duration mechanical stimuli [[28], [29], [30]]. The results herein suggest that the SF may support the maintenance of the cartilage extracellular matrix by replenishing amino acids to the chondrocytes in response to mechanical stimuli.

Acute exercise, defined here as one night of voluntary wheel running, stimulated both inflammatory and anti-inflammatory pathways in mouse SF. Prostaglandin biosynthesis was downregulated after exercise, and a prostaglandin derivative was identified as a significantly depleted mechanosensitive metabolite. Given the pro-inflammatory role of prostaglandins, the observed decrease in the intensity of metabolites involved in prostaglandin biosynthesis may be an anti-inflammatory effect of acute exercise. Other inflammation-related pathways altered with acute exercise included citrulline-nitric oxide cycle and catecholamine biosynthesis. Nitric oxide regulates blood flow and plays an anti-inflammatory role under physiologically normal conditions, and catecholamines can both elicit and attenuate inflammatory responses during exercise [31,32]. These results suggest that acute exercise may stimulate both inflammatory and immunosuppressive effects, consistent with previous findings [31,33]. The emerging paradigm of OA research incorporates several biological processes beyond mechanical wear including inflammation in the synovium, articular cartilage, and SF [34,35]. Inflammation is also associated with OA risk factors including joint trauma, age, and obesity [36]. This may partially explain the importance of exercise in managing inflammation-associated OA pain and damage in the joint.

Ubiquinol, an endogenous antioxidant, was significantly upregulated after acute exercise. Recent evidence suggests that oxidative stress in the joint damages the surrounding tissue and can lead to increased OA severity [37,38]. Regulation of oxidative stress in chondrocytes has been of particular interest for therapeutic targets to slow OA progression [38]. Further studies are needed using an OA mouse model to determine if acute exercise reduces oxidative stress-associated damage to the joint by upregulating antioxidants, such as ubiquinol. This may provide a mechanistic explanation for the use of exercise as a therapeutic intervention to manage OA.

Acute exercise significantly altered a variety of phospholipids. PC, PS, and PA were identified as mechanosensitive metabolites, and fatty acid biosynthesis was significantly upregulated after acute exercise. These data are from metabolite features detected in the synovial fluid, so cytosolic responses could differ. The mechanical function of SF is to lubricate articular cartilage surfaces. The major components of SF that contribute to its boundary lubrication function include lubricin, hyaluronan, and membrane phospholipids [39]. Previous studies show that altered composition and concentration of lubricating agents in SF are associated with damaged articular cartilage surfaces in both OA and RA [40]. Specifically, the composition of phospholipid species and their concentrations are altered in OA [41,42]. Exercise may play a role in maintaining the composition and concentration of SF phospholipids to support the SF's function as a boundary lubricant. Prior targeted studies have examined the plasma and serum metabolomic response to exercise [[43], [44], [45]]. While there are many similarities between these studies, one differences is that Nayor et al. find evidence for exercise-induced lipolysis in the circulation [43]. In contrast, this study found evidence for fatty acid biosynthesis within the synovial fluid after exercise. Exercise is known to induce substantial changes in lipid metabolism [45], and further studies involving different durations and intensities of exercise, as well as paired serum and synovial fluid analysis, are needed to determine if the observed fatty acid biosynthesis is unique to synovial fluid.

While this study illustrates important metabolomic responses to acute exercise, some limitations apply. It is likely that some of the identified mechanosensitive metabolites are involved in the systemic effects of exercise derived from the circulation. Future studies involving paired serum and SF analyses are required to discriminate these two categories of metabolites. Further, the selection of metabolites using arbitrary thresholds (fold change >2, VIP scores >1.5, correlation >0.7 or ​< ​0.7) may affect the pathway analysis outcomes. PLS-DA includes the risk of overfitting which may also affect these results. Global metabolomic profiling by LC-MS is beneficial for quantifying the physiological state of a biological system, but it cannot identify all metabolite features due to the lack of comprehensive metabolite databases and standards libraries. Future studies will employ additional targeted metabolomics methods to confirm putative identities of metabolites in pathways of interest. Conducting this study in mice provides an additional limitation as the small size of the knee joint provides a limited volume of synovial fluid that is difficult to collect and accurately quantify. This difficulty likely increases the variance of metabolite measurements. Additionally, despite the physiologic benefits of using voluntary wheel running as an exercise stimulus, the dose and intensity of exercise was variable among animals, which also likely contributed to increased variability. Finally, because the mice of this study were young adult aged (14 weeks), these results may not apply to osteoarthritic joints in older patients.

Despite these limitations, this study holds important findings for joint mechanobiology. Global metabolomic profiling can discriminate between acute exercised and control SF, thereby identifying mechanosensitive metabolites and pathways. The results of this study provide several avenues to further explore the beneficial effects of acute exercise on joint metabolism. Specifically, this study suggests that acute exercise stimulates both pro-inflammatory and anti-inflammatory metabolic pathways. Chronic exercise conditions may reveal more beneficial effects on joint metabolism and inflammation. These findings also suggest that exercise may promote joint homeostasis by facilitating amino acid metabolism for protein synthesis and increasing the production of antioxidants to reduce oxidative-stress in joint tissues. This study provides a metabolic phenotype for comparison for future studies that investigate the impact of acute exercise in an OA joint.
